# Supplementary material for: Gene expression patterns of novel visual and non-visual opsin families in immature and mature Japanese eel males
Source: PeerJ. 2020 Feb 27;8:e8326. doi: 10.7717/peerj.8326 (PMC7049458; doi:10.7717/peerj.8326)
Supplement: Table S1 [file peerj-08-8326-s001.docx]

**Table 1. Primer sets in this study**

| **Gene ID** | **Oligo ID** | **Sequence** | **Product size (bp)** |
| --- | --- | --- | --- |
| EF1–alpha | Forward | 5–TCACCCTGGGAGTAAAGCAG–3 | 222 |
|  | Reverse | 5–TCCATCCCTTGAACCAGGAC–3 |  |
| Opn4x1 | Forward | 5–GGATCACCTCCATGATCACC–3 | 189 |
|  | Reverse | 5–GGCCCTCTGGAATGTATGAA–3 |  |
| Opn4x2 | Forward | 5–GAGTGGGTGTTCGGTGAACT–3 | 191 |
|  | Reverse | 5–GAGTACAGCCACACCAGCAG–3 |  |
| Opn4m1 | Forward | 5–AATTCCACCGCATGAAGAAC–3 | 197 |
|  | Reverse | 5–GATTATGGGGTTGTGGATGG–3 |  |
| Opn4m2 | Forward | 5–ACTGCAACGGGACATTTAGG–3 | 226 |
|  | Reverse | 5–CAGAACGCGTAGATCACCAG–3 |  |
| Peropsin | Forward | 5–ATGTCTGTGATTGCGGTGAA–3 | 212 |
|  | Reverse | 5–AGGCACACAATGGAGTAGGG–3 |  |
| Opn5–like | Forward | 5–CCAGCCGAGTTCTTCATTGT–3 | 177 |
|  | Reverse | 5–TGTGAGGTTGGTCAGACTGC–3 |  |
| Opn5 | Forward | 5–GCCTCCAAATTGTCGAAAGA–3 | 191 |
|  | Reverse | 5–GGCTTCCCTGTGACTGTGAT–3 |  |
| Opn3 | Forward | 5–TTGCCTTCACTATCGGAACC–3 | 194 |
|  | Reverse | 5–TATCCACCCTCCTTTGATGC–3 |  |
| TMT1 | Forward | 5–TTGGAACTCCGTTCAGCTTT–3 | 161 |
|  | Reverse | 5–GGAGGCCATCATGGTACAGT–3 |  |
| TMT2 | Forward | 5–GCTGGGCTGGAGTAGTTACG–3 | 187 |
|  | Reverse | 5–GATCTTCCCCACCTGTTTGA–3 |  |
| TMT3 | Forward | 5–TTCGTCTTCTGCCTGTTCCT–3 | 161 |
|  | Reverse | 5–AGCAGGTAGCAGGACACCAT–3 |  |
| VA–opsin | Forward | 5–CAGCTACACCACCAGCAAGA–3 | 205 |
|  | Reverse | 5–CGGTTTCCTGGCATTACCTA–3 |  |
| Parapinopsin | Forward | 5–CTGGAGGGGGTAAAGACCTC–3 | 227 |
|  | Reverse | 5–ACAATTACCATGCGGGCTAC–3 |  |
| SWS2 | Forward | 5–AGATGGTGGTGGTGATGGT–3 | 169 |
|  | Reverse | 5–GACGTAGATGACGGGGTTG–3 |  |
| Rh2 | Forward | 5–CACCCAGAAAGCAGAGAAGG–3 | 173 |
|  | Reverse | 5–ACGCTGAGCTCTTGGAGAAG–3 |  |
| Exo–Rhod | Forward | 5–GTGGCTGACCTCTTCATGGT–3 | 189 |
|  | Reverse | 5–CACAGGCTTGCAGACCACTA–3 |  |
| DSO | Forward | 5–TCACCATCGAGCACAAGAAG–3 | 206 |
|  | Reverse | 5–AACCAGAGACCAGAGCGAAA–3 |  |
| FWO | Forward | 5–CGATGTACACCTCCATGCAC–3 | 185 |
|  | Reverse | 5–CATGATGGCATGGTTCTCAC–3 |  |
